# *Trichomonas* Infection in a Community of Free-Ranging Domestic and Wild Columbiformes and Bonelli's Eagle (*Aquila fasciata*)

**DOI:** 10.3389/fvets.2019.00148

**Published:** 2019-05-29

**Authors:** Nuno Santos, José Jambas, António Monteiro, Jorge Amaral, Nuno Martins, Javier Garcia, Ana Martinez Fernández, Kevin Morris Tyler, Tereza Almeida, Joana Abrantes, Pedro J. Esteves

**Affiliations:** ^1^CIBIO/InBio—Research Center in Biodiversity and Genetic Resources, Universidade do Porto, Vairão, Portugal; ^2^Oriolus Ambiente Ecoturismo Lda, Atenor, Portugal; ^3^Parque Natural do Douro Internacional, Instituto de Conservação da Natureza e Florestas I.P., Mogadouro, Portugal; ^4^Palombar—Associação para a Conservação da Natureza e do Património Rural, Uva, Portugal; ^5^Department of Biodiversity and Environmental Management, University of León, León, Spain; ^6^Servicio Territorial de Medio Ambiente, Junta de Castilla y León, Zamora, Spain; ^7^Biomedical Research Centre, Norwich Medical School, University of East Anglia, Norwich, United Kingdom; ^8^Departamento de Biologia, Faculdade de Ciências, Universidade do Porto, Porto, Portugal

**Keywords:** *Trichomonas gallinae*, Columbiformes, *Aquila fasciata*, *Streptopelia turtur*, *Columba livia*, multi-host-pathogen system, wildlife conservation

## Abstract

*Trichomonas* gallinae is a pathogen of conservation relevance, whose main maintenance hosts are Columbiformes, but spillover to avian predators has been described. The goal of this study was to characterize the epidemiology of *Trichomonas* spp. in a community of free-ranging domestic and wild Columbiformes and an endangered predator, Bonelli's eagle *Aquila fasciata*. We surveyed 253 live-captured Rock doves, 16 nestling Bonelli's eagles and 41 hunted Columbiformes. Oro-esophageal swabs were incubated in culture media and *Trichomonas* spp. isolated from Bonelli's eagle (6.3%, CI_95_ 1.1-28.3), Turtle dove *Streptopelia turtur* (56.3%, CI_95_ 39.3–71.8), Wood pigeon *Columba palumbus* (83.3%, CI_95_ 43.7–97.0) and Rock dove *Columba livia* (68.4%, CI_95_ 62.4–73.8). Infected Rock doves showed significantly poorer body condition than uninfected ones (*p* = 0.022). From a subset of 32 isolates, 18S and ITS1/5.8S/ITS2 rRNA genes were sequenced and Maximum-Likelihood trees inferred. Four ribotypes of *Trichomonas* spp. were identified. In this study area *Trichomonas* spp. seem to persist in a multi-host system involving several species of Columbiformes. Conservation actions aimed at increasing the availability of trophic resources for Bonelli's eagles through Rock dove restocking should consider the risk of pathogen transmission and of introduction of alien strains.

## Introduction

Trichomonosis is an avian disease of conservation relevance, caused by infection with *Trichomonas gallinae* (1–3). Typical lesions in the upper digestive tract range from mild inflammation to caseous masses that can partially obstruct the digestive or respiratory tracts and progress to systemic disease (3). Trichomonosis can be present as subclinical to subacute, depending on the host species, immune status and the pathogenicity of the strain (1, 2, 4–8). Transmission can be direct, such as through “crop milk” or consumption of infected prey or indirect such as through contaminated water (3, 9–11).

*Trichomonas gallinae* is an important pathogen of Columbiformes, Falconiformes, and Passeriformes (3). It has been implicated in the decline of Band-tailed pigeons (*Patagioenas fasciata*) in western USA (2) and a highly pathogenic strain has recently emerged in North-Western Europe, causing population declines of finch species (3, 7, 12). Columbiformes are maintenance hosts for *T. gallinae* and the Rock dove (*Columba livia*) is considered the natural host of this pathogen (3). All species of Columbiformes occurring in Europe have been shown to harbor endemic *Trichomonas gallinae* infection (8, 13, 14). Particularly the Turtle dove (*Streptopelia turtur*) was shown to be heavily infected by *Trichomonas gallinae* (8, 15). European populations of Turtle doves heavily declined in the last decades and their migration routes pass through the Iberian Peninsula (16), with the potential of disseminating new strains. On the other hand, raptors are considered spillover hosts, acquiring infection from infected prey (3, 17).

Bonelli's eagle (*Aquila fasciata*) is a conservation priority species in Europe because of widespread declines in the last decades of the 20th century (18, 19). Most of the European population inhabits the Iberian Peninsula where its diet is based on Columbiformes, Red-legged partridge (*Alectoris rufa*) and Wild rabbit (*Oryctolagus cuniculus*) (20–22). Marginal populations of this species were shown to have low genetic variability (23), which could make them more vulnerable to emerging pathogens (24). Trichomonosis was shown to kill 2–14% of nestling Bonelli's eagles in several Iberian populations (25, 26). A marginal population of Bonelli's eagle comprising 22–24 pairs breeds in the *Douro Internacional/Arribes del Duero* natural parks and adjoining areas in northwestern Iberian Peninsula (19, 27, 28). This population is characterized by a low productivity, which has been linked to food shortage (28). Conservation management actions include supplementary feeding, either direct or through restocking of traditional pigeon lofts with Rock doves (27, 29).

**Figure 1:**
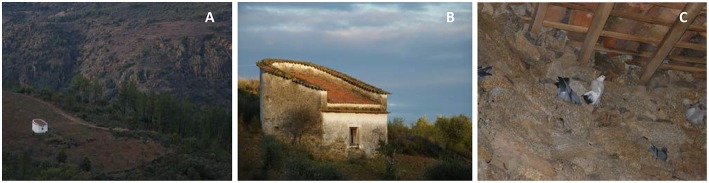
Images of traditional pigeon lofts. **(A)** Landscape view; **(B)** outside view; **(C)** inside with Rock doves *Columba livia*.

Traditional pigeon lofts (TPL) are human constructions typical of Northwestern Iberian Peninsula (Figure 1), scattered across the landscape and originally aimed at producing Rock doves for human consumption and organic fertilizer for crops (30). These buildings provide nocturnal shelter and breeding sites for Rock doves, which are otherwise free-ranging (29, 30). Nowadays most TPL are abandoned but some have been recovered and restocked to increase prey availability for Bonelli's eagles (29).

The aim of this study was to investigate the epidemiology of *Trichomonas* spp. in the community of free-ranging domestic and wild Columbiformes and their endangered avian predator, Bonelli's eagle, in an area of Northwestern Iberian Peninsula. Additionally, we aimed to contribute to elucidating if restocking TPL with Rock doves could increase the exposure of Bonelli's eagles to infection by *Trichomonas* spp.

## Materials and Methods

### Study Area

**Figure 2:**
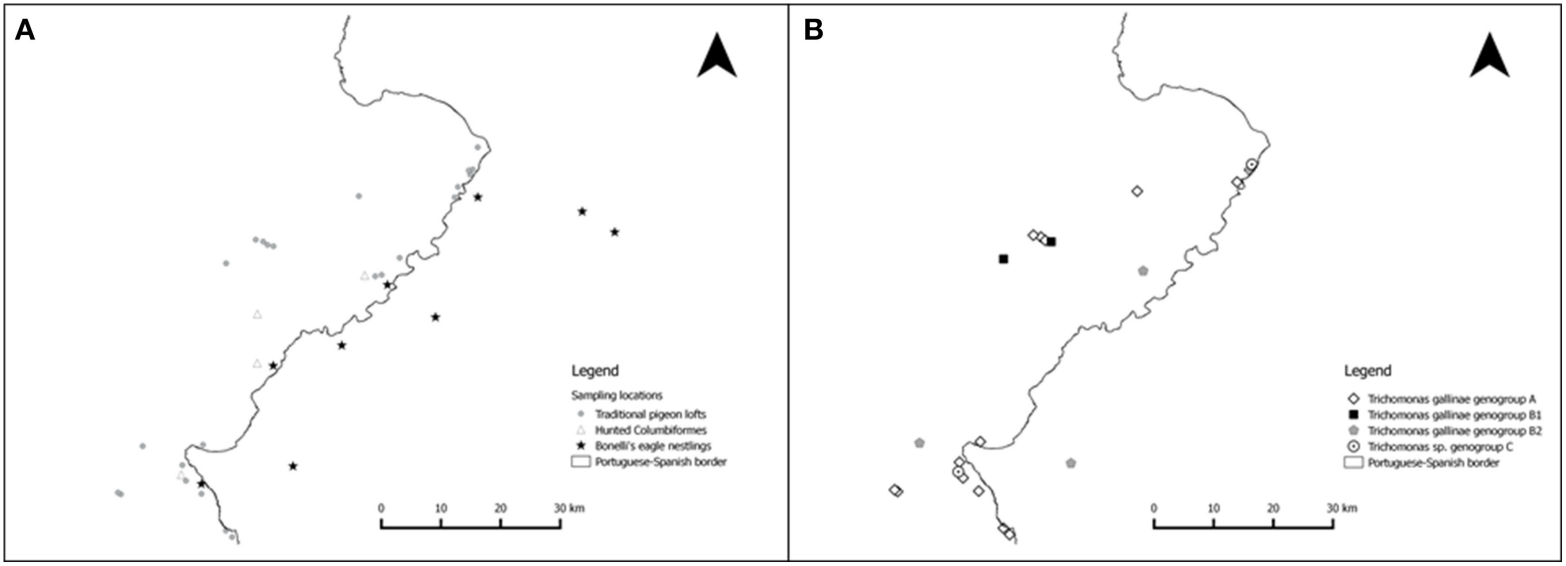
Location of the sample collection sites and of the *Trichomonas* sp. genogroups isolated. **(A)** Sampled traditional pigeon lofts (circles), wild Columbiformes (triangles), and Bonelli's eagle nests (stars). Due to the threatened status of Bonelli's eagles and their sensitivity to disturbance, the location of the sampling sites for this species is not exact. **(B)** Geographical sampling location of the *Trichomonas* sp. genogroups identified in the study area.

The study area consists of *Douro Internacional*/*Arribes del Duero* natural parks (comprising 193,000 hectares) and adjoining areas in Northwestern Iberian Peninsula (centroid 41° 11′ 36′ ′ N, 6° 45′ 49^′′^ W), encompassing both Portuguese and Spanish territory (Figure 2A). The area is an extensive plateau (600–800 m asl) where Douro river and several main tributaries (Águeda, Sabor, Côa, Tormes, and Huebra rivers) carved steep canyons in granite or shale rock. The landscape is highly fragmented, with oak (*Quercus pyrenaica, Q. faginea, Q. rotundifolia*, and *Q. suber*) and juniper (*Juniperus oxycedrus*) woods interspersed with shrub (mainly *Cytisus* sp. and *Cistus* sp.) and agricultural areas (mainly wheat *Triticum* sp., olive *Olea europaea*, vine *Vitis vinifera*, and pasture). The area is included in the Mesomediterranean and Supramediterranean bioclimatic zones showing Mediterranean-Subcontinental climate with large thermal amplitude, hot and dry summers and relatively cool and humid winters (31, 32).

### Sample and Data Collection

From August 2008 to October 2009 we surveyed *Trichomonas* spp. in 253 live-captured Rock doves and 41 hunted Columbiformes, comprising 32 Turtle doves, 6 Wood pigeons (*Columba palumbus*) and 3 Rock doves. Rock doves were live-captured on 24 TPL (Figure 2A) by closing the exits during the night and manually capturing them inside. To prevent repeated sampling of individuals, each TPL was sampled only once and Rock doves were kept in cages and released when all were sampled (<2 h of restraint). Samples from wild Columbiformes were obtained from animals hunted for recreational purposes, according to Portuguese and European legislation. No animals were killed for the purpose of this study. This study was carried out in accordance with national and European legislation and the recommendations of the “Guidelines on the care and use of wildlife” (33).

From May 2014 to June 2016 we surveyed *Trichomonas* spp. in 16 nestling Bonelli's eagles, sampled during regular monitoring for ringing (Figure 2A). Samples were obtained under permits 412/2014 and 316/2016 (*Junta de Castilla y León*, Spain) and 415/2014 (*Instituto de Conservação da Natureza e Florestas*, Portugal).

An oro-esophageal swab was obtained from each bird, immediately inoculated in InPouch™ media (Biomed Diagnostics Inc., White City, OR, USA) and kept at room temperature and away from sunlight until incubation (7–50 h post collection). From individual Rock doves, we collected data on sex, age class, weight, wing length, and plumage (wild-type or other). The residuals of a linear regression between log weight and log wing length of each Rock dove were used as indicators of body condition. The presence of macroscopic lesions compatible with trichomonosis was systematically investigated in the Bonelli's eagle nestlings but not in the Columbiformes.

Data were collected on each TPL during the sampling procedure and included geographical location, number of Rock doves, presence of other avian species, provision of water, location within occupied Bonelli's eagle territories (<3 km straight line from the center of a territory occupied by a pair during the previous breeding season) and the interval between collection and incubation of the biological samples.

### Laboratory Analysis

Culture media were incubated at 37°C for 6 days and isolation of *Trichomonas* spp. was assessed by daily bright field microscopy at 100x magnification and considered positive when motile, flagellated protozoans were present in the culture media. Media containing *Trichomonas* were centrifuged at 1,430 × g for 10 min, the supernatant discarded, and the pellet re-suspended in PBS and stored at −20°C. DNA was extracted from a subset of 32 *Trichomonas* isolates, 1–2 per host species and TPL studied, with DNeasy Blood & Tissue Kit (Qiagen) and purified with Exo-AP Clean-up (Thermo Scientific), according to the manufacturer's instructions. For amplification of the 18S and ITS1/5.8S/ITS2 rRNA genes, previously established primers HM-LONG-F [5' AGGAAGCACACTATGGTCATAG 3'; (34)] and TFR1 [5' TGCTTCAGTTCAGCGGGTCTTCC 3'; (35)] were used. PCR amplification was performed with a PCR Master Mix (Phusion). Cycling parameters consisted of an initial denaturation at 98°C for 3 min, followed by 45 cycles of 98°C for 30 s, 61°C for 30 s and 72°C for 1 min, and 72°C for 5 min for the final extension step. products were purified and sequenced on an automatic sequencer PRISM 310 Genetic Analyzer (PE Applied Biosystems, Foster City, CA, USA) with the amplification primers and internal primer HM-LONG-R [5' CGTTACCTTGTTACGACTTCTCCTT 3'; (34)]. The sequences were deposited in GenBank under the following accession numbers: MK932769-773, MK932775-777. The ribotype nomenclature proposed by Gerhold et al. (36) and Grabensteiner et al. (37) is used throughout this paper.

### Statistical Analysis

Differences in prevalence between TPL were analyzed by non-parametric methods (Kruskal-Wallis test). We performed a binomial GLM with *Trichomonas* spp. infection status in individual Rock doves as dependent variable and individual traits (age class, sex, body condition, and plumage type) as independent variables (Table 1). We performed another GLM with *Trichomonas* spp. prevalence in TPL as dependent variable and environmental and sampling features as independent variables (number of Rock doves, presence of other avian species and water availability inside the TPL, location within an occupied Bonelli's eagle territory, and interval from collection of samples to start of incubation) (Table 1). We considered all TPL <3 km straight distance from an occupied nest as being located within an occupied territory, based on the home ranges of breeding Bonelli's eagles in the Iberian Peninsula (38, 39). Collinearity was checked by calculating the Variance Inflation Factor with a threshold of 4 and goodness of fit assessed by calculating the Hosmer-Lemeshow χ^2^. Statistical analyses were performed in R-3.3.2 (R Development Core Team). Maps were produced in QGIS 2.18.0 (QGIS Development Team). Maximum-Likelihood (ML) trees were inferred for the ITS1/5.8S/ITS2 and 18S rRNA regions (Table 3) in MEGA6 (40) under the best-fit nucleotide substitution model determined by the same software. Node support was determined from 500 bootstrap replicates of the ML trees.

**Table 1:** Summary of the independent variables included in the statistical analysis.

**Sampling unit**	**Independent variable**	**Type**	***n***	**Average (SD)**	**Categories**
Rock dove	Age class	Categorical			125 adults 55 juveniles 73 n.a.
	Sex	Categorical			62 females 75 males 116 n.a.
	Weight	Continuous	126	309.9 g (69.4)	
	Wing length	Continuous	127	221.9 mm (14.8)	
	Plumage class	Categorical			41 wild-type 93 other 119 n.a.
	Interval from collection to incubation	Continuous	24	27.2 h (12.8)	
Traditional pigeon loft	Number of rock doves	Count	24	51.9 (34.4)	
	Other avian species	Categorical	24		17 no other species 7 other species^a^
	Provision of water	Categorical	24		12 yes 12 no
	Within occupied Bonelli's eagle territory (<3 km from the nest site)	Categorical	24		10 within 14 outside
	Interval from collection to incubation	Continuous	24	27.2 h (12.8)	

a *Sturnus unicolor, Tyto alba*.

*n.a., not available*.

## Results

### Descriptive Epidemiology

**Figure 3:**
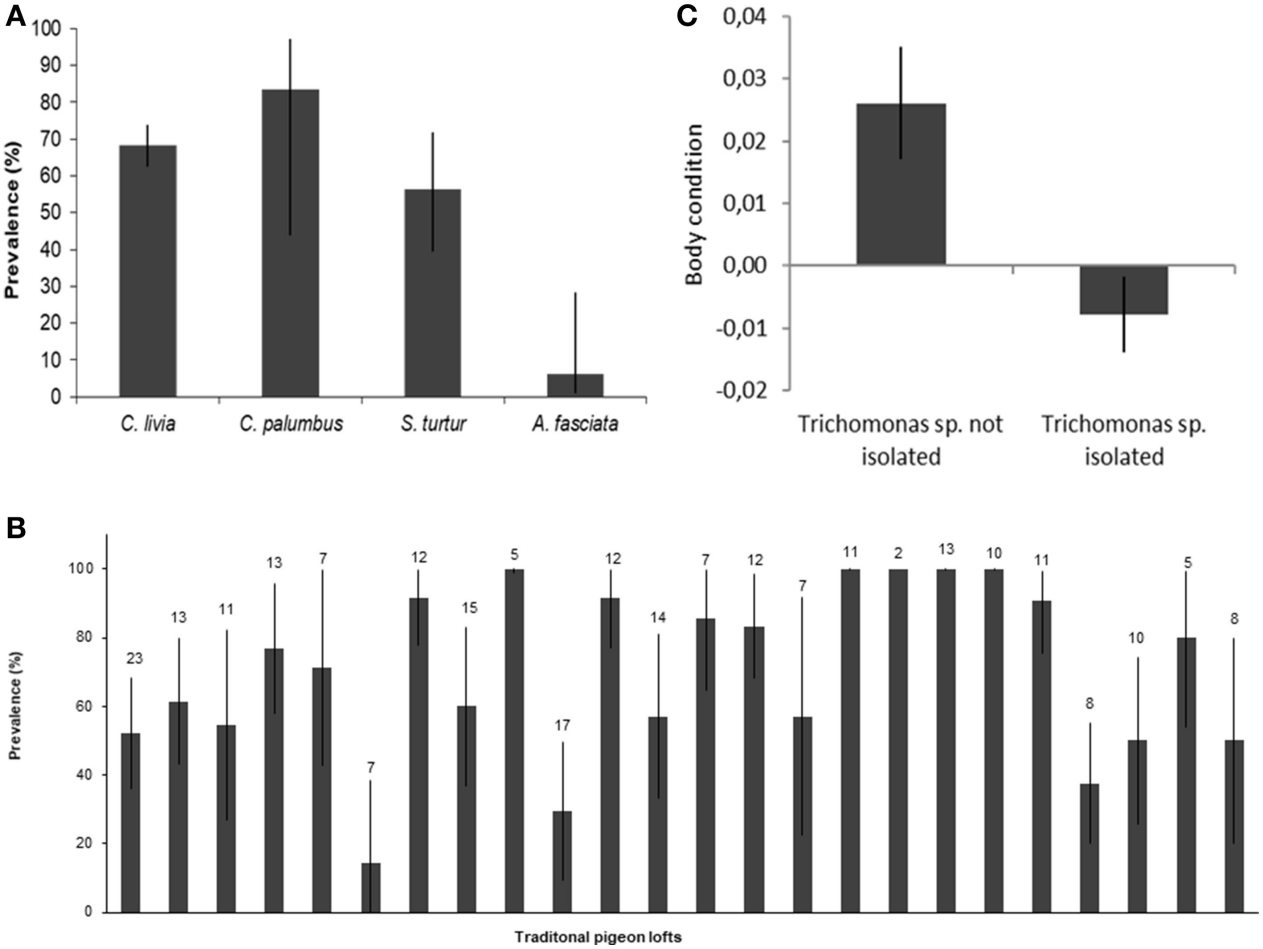
Determinants of *Trichomonas* sp. infection. *Trichomonas* sp. prevalence with 95% confidence intervals by **(A)** species; **(B)** traditional pigeon loft, with the sample size for each TPL; **(C)** Rock dove body condition by *Trichomonas* sp. infection status, with standard error of the mean.

*Trichomonas* spp. were isolated from 1/16 (6.3%, CI_95_ 1.1–28.3) nestling Bonelli's eagles, 18/32 Turtle doves (56.3%, CI_95_ 39.3–71.8, *n* = 32), 5/6 Wood pigeons (83.3%, CI_95_ 43.7–97.0), and 175/256 Rock doves (68.4%, CI_95_ 62.4–73.8) (Figure 3A). Prevalence of *Trichomonas* spp. in TPL ranged 14.3–100% (Figure 3B), showing statistically significant differences between TPL (*p* < 0.001, Kruskal-Wallis test).

### Determinants of Infection

In the binomial GLM with *Trichomonas* spp. isolation in individual Rock doves as dependent variable (Hosmer-Lemeshow χ^2^ = 6.445, *p* = 0.598), the only significant determinant of infection was the body condition, which was significantly lower (*p* = 0.021) in infected Rock doves (Table 2 and Figure 3C). In the GLM with *Trichomonas* spp. prevalence in TPL as dependent variable (Hosmer-Lemeshow χ^2^ = −1.2273, *p* = 1), no significant determinants of prevalence of infection were identified (Table 2).

**Table 2:** GenBank entries used in the phylogeny of the ITS1/5.8S/ITS2 and 18S rRNA regions.

**Species**	**Host**	**Origin**	**ITS1/5.8S/ITS2**	**18S rRNA**
*Trichomonas* sp.	Pied imperial-pigeon	Australia	JQ755285	JQ027618
	*Ducula bicolor*			
*Trichomonas* sp.	Chestnut-quilled Rock-pigeon	Australia	JQ755286	JX512966
	*Petrophassa rufipennis*			
*Trichomonas* sp.	Racing pigeon	Austria	FN433473	FN433486
	*Columba livia*			
*Trichomonas* sp.	Pied imperial-pigeon	Australia	JQ027616	JQ027618
	*Ducula bicolor*			
*Trichomonas* sp.	Southern crowned pigeon	Papua New Guinea	JX512967	JX512959
	*Goura scheepmakeri*			
*Trichomonas* sp.	New Zealand pigeon	New Zealand	JQ692126	n/a
	*Hemiphaga novaeseelandiae*			
*Trichomonas* sp.	New Zealand pigeon	New Zealand	JQ692128	JQ692127
	*Hemiphaga novaeseelandiae*			
*Trichomonas* sp.	Pheasant pigeon	Papua New Guinea	JX512969	JX512961
	*Otidiphaps nobilis*			
*Trichomonas* sp.	Turtle Dove	Spain	KX459488	n/a
	*Streptopelia turtur*			
*Trichomonas* sp.	Turtle Dove	Italy	KX459509	n/a
	*Streptopelia turtur*			
*Trichomonas canistomae*	Dog	Czech Republic	AY244652	AY247748
*Trichomonas gallinae*	Broad-winged hawk	USA	EU215368	EU215375
	*Buteo platypterus*			
*Trichomonas gallinae*	Budgerigar	Austria	FN433476	FN433484
	*Melopsittacus undulatus*			
*Trichomonas gallinae*	Wood Pigeon	Germany	KX459442	n/a
	*Columba oenas*			
*Trichomonas gallinae*	Rock Dove	USA	EU215364	EU215373
	*Columba livia*			
*Trichomonas gallinae*	Eurasian collared-dove	USA	EU215364	EU215374
	*Streptopelia decaocto*			
*Trichomonas gallinae*	Rock Dove	Austria	FN433475	FN433480
	*Columba livia*			
*Trichomonas gallinae*	Eurasian collared-dove	Austria	FN433475	FN433482
	*Streptopelia decaocto*			
*Trichomonas* sp.	Common bronzewing	Australia	JQ755275	JQ030999
	*Phaps chalcoptera*			
*Trichomonas gallinae*	Turtle Dove	Malta	KX844988	n/a
	*Streptopelia turtur*			
*Trichomonas tenax*	Human	USA	TTU86615	D49495
*Trichomonas vaginalis*	Human	USA	L29561	TVU17510
*Trichomonas* sp.	Rose-crowned fruit-dove	Australia	JQ755274	JX512962
	*Ptilinopus regina*			
*Trichomonas* sp.	Ornate fruit-dove	Australia	JX512968	JX512960
	*Ptilinopus ornatus*			
*Tetratrichomonas sp*.	Snapping turtle	Czech Republic	AY245133	AY245121
	*Macrochelys temminckii*			
*Pentatrichomonas hominis*	Human	Slovakia	AY245137	AF124609

**Table 3:** Results of the generalized linear models. Analysis of the potential determinants of *Trichomonas* sp. isolation in individual Rock doves and of prevalence in traditional pigeon lofts.

**Dependent variable**	**Independent variable**	**Estimate**	**Statistic**	***p***
*Trichomonas* sp. isolation in individual Rock doves (binomial)	Intercept	2.081	2.625	0.009
	Age class (juveniles)	−0.609	−0.703	0.482
	Sex (male)	−0.509	−0.818	0.413
	Body condition	−11.994	−2.304	0.021
	Interval collection to incubation	−0.020	−0.913	0.361
*Trichomonas* sp. prevalence in traditional pigeon lofts (proportion)	Intercept	100.068	5.923	< 0.001
	Number of Rock doves	−0.025	−0.142	0.888
	Other avian species	13.017	1.204	0.244
	Provision of water	−11.801	−1.027	0.318
	Within occupied Bonelli's eagle territory	−4.616	−0.457	0.653
	Interval from collection to incubation	−0.896	−1.906	0.073

### Molecular Epidemiology

**Figure 4:**
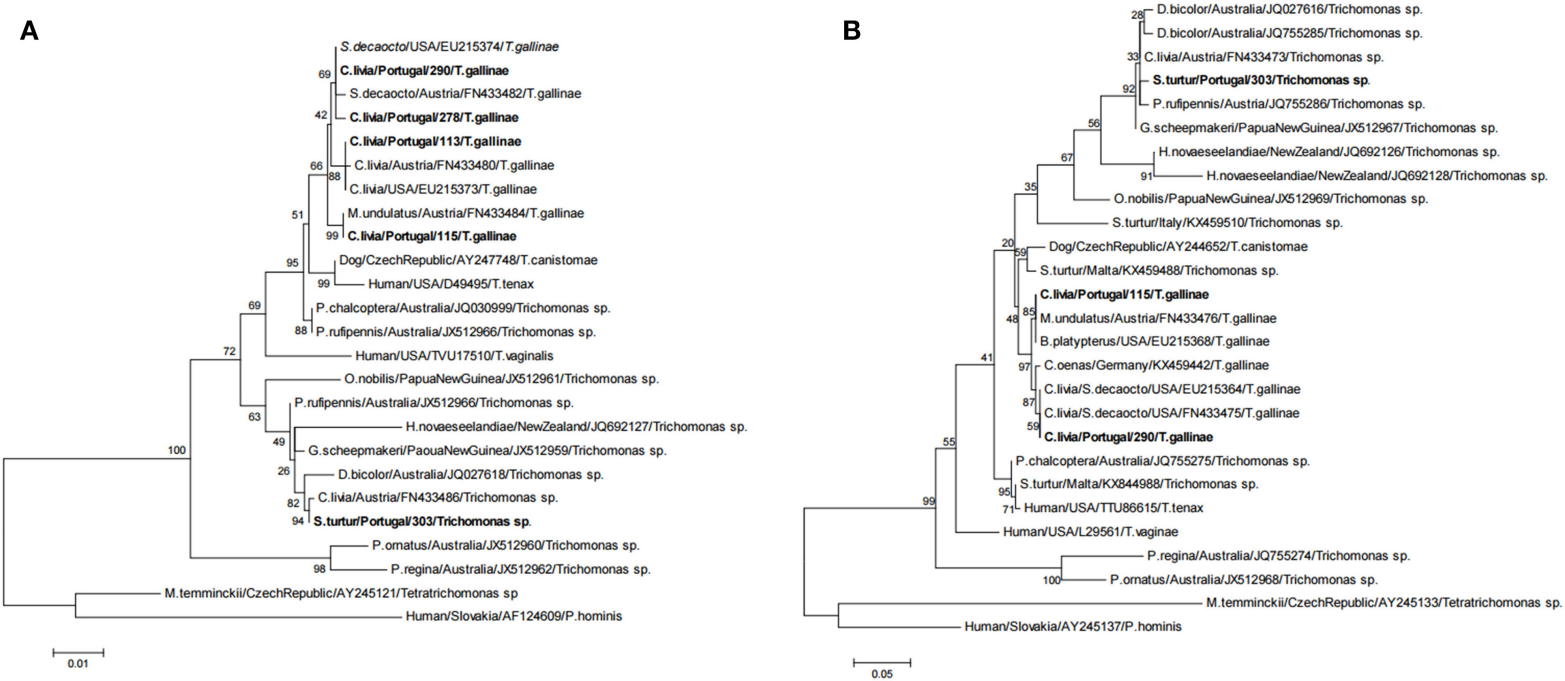
Phylogenetic tree of the *Trichomonas* isolates based on **(A)** 18S rRNA (1155bp) and **(B)** ITS1/5.8S/ITS2 (347bp). The phylogenetic analyses were estimated using the Maximum Likelihood (ML) method available in MEGA6 (40) under the best-fit nucleotide substitution model determined by the same software. Node support was determined from 500 bootstrap replicates of the ML trees. GenBank accession numbers of the retrieved sequences are indicated.

Three ribotypes of *Trichomonas gallinae* and one ribotype of *Trichomonas* sp. were obtained from the 32 isolates that were sequenced (Table 4 and Figure 4). *Trichomonas gallinae* ribotype 18S-VI, ITS-IV/B (*n* = 23) was distributed in TPL throughout the study area, while *T. gallinae* ribotype 18S-IV, ITS-I/D (*n* = 3) was detected in 2 TPL recently restocked with Rock doves acquired from a mixed-species avian collection. Both were only detected in Rock doves. *Trichomonas gallinae* ribotype 18S-II, ITS-I/D (*n* = 4) was detected in Rock doves from 2 TPL, 1 Wood pigeon and 1 nestling Bonelli's eagle, throughout the study area. Ribotype 18S-VIII, ITS-III of *Trichomonas* sp. (*n* = 3) was detected in 2 Rock doves from a single TPL and in 1 hunted Turtle dove in the northern and southern parts of the study area, respectively (Table 4 and Figure 2B).

**Table 4:** Ribotypes of *Trichomonas* sp. identified in our sample based on ITS1/5.8S/ITS2 and 18S RNA sequences. Number of the isolates per species and traditional pigeon lofts (TPL). Ribotype nomenclature follows Gerhold et al. (36) and Grabensteiner et al. (37).

**Ribotype**	**Host species**	**Number of isolates sequenced**	**Number of TPL**	**Representative isolates**
18S-VI, ITS-IV/B	*C. livia*	22	14	*C. livia*/Portugal/115/*T. gallinae*
18S-IV, ITS-I/D	*C. livia*	3	2	*C. livia*/Portugal/290/*T. gallinae*
				*C.livia*/Portugal/278/*T. gallinae*
18S-II, ITS-I/D	*C. livia*	2	2	*C. livia*/Portugal/113/*T. gallinae*
	*C. palumbus*	1		
	*A. fasciata*	1		
18S-VIII, ITS-III	*C. livia*	2	1	*S. turtur*/Portugal/303/*Trichomonas* sp.
	*S. turtur*	1		

## Discussion

This study reports the epidemiology of *Trichomonas* spp. infection in the community of maintenance hosts for this pathogen and apparent spillover to an endangered avian predator. These results suggest that at least the *Trichomonas gallinae* ribotype 18S-II, ITS-227 I/D (*n* = 4), and ribotype 18S-VIII, ITS-III of *Trichomonas* sp. are maintained in the study area by a multi-host-pathogen system (41), where infection is likely transmitted between several species of free-ranging domestic and wild Columbiformes, as also documented in the United Kingdom (13). The inter-species transmission of *Trichomonas* spp. is supported by the high prevalence of infection in all studied Columbiformes species and particularly by the isolation of two genogroups in multiple species of Columbiformes.

Four distinct ribotypes of *Trichomonas* were detected in Rock doves, two of which are presumed to naturally occur in the study area (*T. gallinae* ribotypes 18S-VI, ITS-IV/B and 18S-II, ITS-I/D), as they were previously reported in this host species elsewhere in Europe (42). On the other hand, *T. gallinae* ribotype 18S-IV, ITS-I/D was previously reported only in Eurasian collared doves (36, 37), this being to our knowledge the first report of its isolation from Rock doves. *Trichomonas gallinae* ribotype 18S-IV, ITS-I/D was detected in the present study in two TPL recently restocked with Rock doves acquired in a mixed species collection. This observation highlights the risk of introducing new pathogen strains in local host communities by means of translocations for conservation purposes (43, 44). Here we report the first isolation in European wild Columbiformes of *Trichomonas* sp. ribotype 18S-VIII, ITS-III. This genogroup was previously detected in Europe only in 2007, in a domestic Rock dove in Austria (37).

Interestingly the most common and widespread *T. gallinae* ribotype 18S-VI, ITS-IV/B was only detected in Rock doves, while the less common *T. gallinae* ribotype 18S-II, ITS-I/D was also detected in Wood pigeon and Bonelli's eagle. Whether this corresponds to a greater ability of the latter for spillover to other host species is still to be determined. Differential inter-species transmission rates have been suggested for other *T. gallinae* genogroups, as well as differential pathogenicity (7, 15). As we only sequenced the 18S and ITS1/5.8S/ITS2 rRNA genes, more genetic diversity could have gone unnoticed in our sample of *Trichomonas* isolates. Furthermore, the opportunistic sampling performed in Bonelli's eagle, due to constraints in accessing nests of this endangered species, means there is no temporal overlap between samples collected from this species and those from Columbiformes.

Trichomonosis can be an important cause of mortality for nestling Bonelli's eagles; nevertheless, subclinical infections are more common than clinical ones (25, 26). In the present investigation only one nestling was shown to harbor *Trichomonas gallinae*, but no lesions were detected during handling in the nest and it survived to fledging. We could not isolate *Trichomonas* from two other nestlings with compatible lesions (oropharyngeal caseous masses), which could be due to a different etiology or a long interval between collection of samples and incubation. Hernández et al. (27) also estimated a low prevalence in this same population (10%). Nevertheless, the low sample size precludes drawing conclusions on the pathogenicity and potential impact of infection by *Trichomonas gallinae* on this Bonelli's eagle population.

Comparable studies in other populations of Bonelli's eagle report higher prevalence in nestlings, which could be due to the low sample size or to the relatively long interval between collection of the samples and incubation in our study. *Trichomonas gallinae* was isolated from 36 to 68.8% of nestlings in 3 populations from Southern Iberian Peninsula (5, 25, 26), where Columbiformes are 28.5–39.2% of the prey items of Bonelli's eagles (20, 45). In our study population, Columbiformes are 20.6% of the diet of this species, with a preponderance of Rock doves over Wood pigeons (27). Given the high prevalence of infection by *Trichomonas gallinae* in all species of domestic and wild Columbiformes and the importance of these prey on the diet of Bonelli's eagles across its Iberian range, most if not all nestlings should be exposed to *Trichomonas* sp. (26). The variable prevalence of infection in nestlings from different populations could be explained by individual, regional and temporal heterogeneities in the pathogenicity of *Trichomonas gallinae* strains present in the local population of Columbiformes or the immune status of Bonelli's eagle nestlings. Marginal Iberian populations of Bonelli's eagle were shown to have low genetic diversity (23), which could impair their immune response (24). Further large-scale studies on this predator-parasite-prey system, addressing the link between diet and *Trichomonas gallinae* prevalence, are needed to elucidate the reasons for this heterogeneity in prevalence between populations of Bonelli's eagles.

One of the drivers of this study was the concern that restocking TPL with Rock doves would increase the exposure of Bonelli's eagles to *Trichomonas gallinae* (46). While this could occur if the consumption of Rock doves increases in response to this conservation action, we show that *Trichomonas* spp. infection is widespread in the wild Columbiformes community. The single case of infection in a Bonelli's eagle nestling took place in a territory where no TPL restocking was carried out. Nevertheless, emerging *Trichomonas* spp. strains might be amplified in TPL, providing opportunity for spillover to Bonelli's eagles. Systematic disease surveillance in TPL should be implemented to detect the potential introduction of new strains and contingency plans prepared to prevent spillover to endangered avian predators.

Turtle doves in our sample showed lower prevalence than those reported elsewhere in Europe [67–100% - (8, 13–15)]. European populations of this species have undergone a sustained decline in the last decades, and trichomonosis has been suggested as contributing for this decline (8, 15, 16). The prevalence we determined in other species are comparable to those reported in the literature: 34–70% in Wood pigeon throughout Europe (8, 13, 14, 47) and 45–79% in Rock doves in Spain (5, 14).

Interestingly we observed that Rock doves from which *Trichomonas* spp. were isolated showed poorer body condition. It was shown in other host-pathogen systems that the physiological costs of immune system activation to fight infection trade-off with other physiological needs, such as reproduction, foraging and growth (48, 49). In our sample this translated into a lower body condition of infected Rock doves, as previously shown to occur in other free-ranging Columbiformes infected with *T*. *gallinae* (15, 47). The lower body condition of infected Rock doves could heighten the risk of predation, potentiating interspecies transmission, but being located within a Bonelli's eagle territory was not found to be a risk factor for prevalence in TPL. Studies including the whole community of avian predators of Rock doves are needed to address this question.

*Trichomonas* spp. seem to persist in the study area in a multi-host ecosystem, where intra- and inter-species transmission likely contribute to maintenance, making it demanding to individualize the role of each host species in the epidemiology of infection (41). More detailed molecular epidemiology tools have the potential to further contribute to unveiling the dynamics of pathogen transmission in this host community. Long-term studies will allow uncovering the origin of infection for avian predators such as the Bonelli's eagle, potentially highlighting ways to control spillover to this endangered species. Meanwhile, conservation actions aimed at increasing the availability of trophic resources for Bonelli's eagles should consider the potential risk of increased disease transmission and minimize the risk of introducing alien *Trichomonas* spp. strains.

## Ethics Statement

This study was carried out in accordance with the recommendations of guidelines on the care and use of wildlife (33), Portuguese and European legislations. The study was conducted under permits 412/2014 and 316/2016 (Junta de Castilla y León, Spain) and 415/2014 (Instituto de Conservação da Natureza e Florestas, Portugal).

## Author Contributions

NS, JJ, AM, JG, JrA, and PE designed the study. NS, JJ, AM, JrA, NM, JG, AF, and TA performed the field and laboratory work. NS, TA, KT, JnA, and PE analyzed data and wrote the manuscript. All authors reviewed the manuscript upon submission.

### Conflict of Interest Statement

The authors declare that the research was conducted in the absence of any commercial or financial relationships that could be construed as a potential conflict of interest.
